# Synergistic Effects of Danshen (Salvia Miltiorrhiza Radix et Rhizoma) and Sanqi (Notoginseng Radix et Rhizoma) Combination in Inhibiting Inflammation Mediators in RAW264.7 Cells

**DOI:** 10.1155/2016/5758195

**Published:** 2016-10-18

**Authors:** Xian Zhou, Valentina Razmovski-Naumovski, Dennis Chang, Chunguang Li, Antony Kam, Mitchell Low, Alan Bensoussan, Kelvin Chan

**Affiliations:** ^1^National Institute of Complementary Medicine (NICM), Western Sydney University, Locked Bag 1797, Penrith, NSW 2751, Australia; ^2^South Western Sydney Clinical School, Faculty of Medicine, University of New South Wales, Sydney, NSW, Australia; ^3^Molecular Biology, Nanyang Technological University, 50 Nanyang Avenue, Singapore 639798; ^4^School of Pharmacy and Biomolecular Sciences, Liverpool John Moores University, James Parsons Building, Byrom Street, Liverpool L3 3AF, UK; ^5^Faculty of Sciences, TCM Division, University of Technology Sydney, Main Campus, P.O. Box 123, Broadway, NSW 2007, Australia

## Abstract

*Aims*. This study aims to investigate the possible synergistic interactions of the Danshen-Sanqi combination on vascular disease via their anti-inflammatory activities.* Methods*. Nine combination ratios of Danshen-Sanqi extracts were screened in the RAW264.7 cell line and their anti-inflammatory effects were examined in lipopolysaccharide- (LPS-) induced nitric oxide (NO), tumor necrosis factor (TNF), and monocyte chemoattractant protein-1 (MCP-1) generation pathways. The interaction between Danshen and Sanqi on each target was analysed using combination index (CI) and isobologram models. Additionally, the anti-inflammatory activities of key bioactive compounds from Danshen and Sanqi were tested using the same models. The compounds from each herb that exerted the most potent activity were combined to evaluate their possible synergistic/antagonistic interactions.* Results*. Danshen-Sanqi 8 : 2 was found to be the optimal ratio and exerted a synergistic effect in inhibiting NO, TNF, and MCP-1 when the concentrations were higher than 1.24, 1.89, and 2.17 mg/mL, respectively. Although dihydrotanshinone I (DT) and ginsenoside Rd (Rd) from Danshen and Sanqi, respectively, exhibited the greatest individual bioactivity in the assays, antagonistic effects were observed for the DT-Rd combination 7 : 3.* Conclusion*. This study provided scientific evidence to support the traditional use of the Danshen-Sanqi combination for vascular disease through their synergistic interactions on anti-inflammatory pathways.

## 1. Introduction 

Vascular disease is a subgroup of cardiovascular diseases, contributing to the leading cause of illness, disability, and death worldwide. It describes an abnormal condition of the blood vessels (arteries and veins) in the tissues or organs mainly induced by vascular dysfunction [1]. Hypertension, obesity, diabetes, high cholesterol levels, stress, and elevated plasma homocysteine are all risk factors which could accelerate the formation of vascular pathological changes [2]. The development of vascular disease is complex and involves interplay of various factors and signaling pathways and the endothelial dysfunction is considered to be an early marker of the disease [3]. Mounting evidence has suggested that proinflammatory responses induced by cytokines and chemokines play a pivotal role in endothelial impairment, leading to endothelial dysfunction, vascular inflammation, and the development of many inflammatory vascular diseases [4–6].

As a major mediator of endothelium-dependent relaxation, nitric oxide (NO) is primarily generated by endothelial nitric oxide synthase (eNOS) under normal conditions and is essential in maintaining the healthy vascular homeostasis between vasodilation and vasoconstriction [7]. Under pathological conditions, however, NO is produced by inducible nitric oxide synthase (iNOS) in response to inflammatory mediators such as lipopolysaccharide (LPS). Its increased expression has been associated with nitrosative stress and impaired vascular function in inflammatory vascular diseases [8]. Tumor necrosis factor (TNF), a major inflammatory mediator, has direct impact on the downregulation of eNOS expression, which impairs endothelium-dependent, NO-mediated vasodilation [9–11]. It is also linked with other risk factors for vascular disease including lipid metabolism, normal aging, and high-fat and high-carbohydrate diets [7]. For atherosclerotic vascular disease, it has been reported that monocyte chemoattractant protein-1 (MCP-1) contributes to the triggering of atherosclerotic plaque growth and rupture. Its increased expression leads to the upregulation of adhesion molecules, which aggravates the formation of atherosclerotic lesions and thrombin [12]. Therefore, the suppression of proinflammatory agents such as iNOS-induced NO, TNF, and MCP-1 represents a potential biological target for the management of inflammatory vascular diseases [13].

It is well recognised that combinational drug therapy is an ideal treatment for vascular diseases due to its complex aetiology and pathophysiology. As combinational drug therapy has become a core treatment strategy for other complex diseases such as cancer, diabetes, and HIV infection, significant progress has been made in the development of mathematical and computational models to facilitate the determination of the synergism of the drugs. Combination index (CI) and isobolograms are well-accepted and practical methods for the analyses of the synergistic/antagonistic interactions of combinational agents acting on the same target/receptor [14]. Traditional Chinese medicine (TCM) employs multiherbal formulations for the prevention and treatment of various diseases, with a concept that the multiple bioactive components in the herbal formula can exert synergistic actions leading to enhanced therapeutic effects. Salvia Miltiorrhiza Radix et Rhizoma (Bunge, Lamiaceae, known as “Danshen” in TCM) and Notoginseng Radix et Rhizoma (Burkill, F. H. Chen, Araliaceae, known as “Sanqi” in TCM) are two of the most popular and well-studied Chinese herbs. In particular, the paired herb combination has been clinically used for years and is officially listed in the Chinese Pharmacopoeia (as Compound Danshen Tablet) with an indication for coronary heart disease [15].

As single herbs, the effects of Danshen and Sanqi have been well studied. For example, a clinical study in patients with hypercholesterolaemia revealed that a Danshen extract produced protective effects in endothelial progenitor cells (EPCs) at least partially* via* reducing the expression of IL-6 and TNF, highlighting its anti-inflammatory activity [16]. Results from previous studies in LPS-stimulated macrophage cells have shown that lipid-soluble compounds of Danshen including tanshinone I (T1), cryptotanshinone I (CT), dihydrotanshinone I (DT), and tanshinone IIA (TIIA) reduce iNOS-induced NO, TNF, IL-1*β*, PGE_2_, and MCP-1 levels [17–19].* Panax notoginseng* saponins (PNS) have been shown to exert antiatherogenic effect through their anti-inflammatory activity in an apolipoprotein E- (apoE-) deficient mouse model and to produce dose-dependent inhibition of MCP-1 mRNA expressions in a high-fat and chronically inflamed induced atherosclerotic rabbit model [20, 21]. Additionally, PNS attenuated atherosclerosis progression partially by suppressing the release of MCP-1 in LPS-stimulated macrophage cells [20]. Ginsenoside Rg1 (Rg1) and ginsenoside Re (Re), two other key bioactive components of Sanqi, have also been shown to reduce LPS-stimulated cytokines such as TNF* in vitro* models [22–24].

To date, however, little is known about the interactions between the two herbs and the evidence for the combined usage has not been elucidated. Therefore, this study aims to investigate the combinational effects of Danshen-Sanqi pair on proinflammatory mediators including TNF, NO (induced by iNOS), and MCP-1 to elucidate their endothelial protection properties in RAW264.7 macrophages cell model. Possible synergistic interactions between the key bioactive components from each herb were analysed using CI and isobologram models.

## 2. Materials and Methods

### 2.1. Preparation of Herbal Samples and Their Chemical Compounds

Crude Danshen and Sanqi herbal materials were sourced from PuraPharm International Ltd., Hong Kong. The raw herbal materials were authenticated by Professor Si-bao Chen from the Department of Applied Biology and Chemical Technology, the Hong Kong Polytechnic University, Hong Kong, China, according to the Hong Kong Materia Medica Standards. The aqueous extracts of Danshen (DS) and Sanqi (SQ) were prepared as follows: 1 g of ground Danshen/Sanqi crude herbal powder (30-mesh size) was weighed and soaked with 30 mL water for 0.5 hours, followed by refluxing with boiling water for another 1 hour. The solution was then centrifuged at 3000 rpm for 5 min and the supernatant was separated and evaporated to dryness using a freeze dryer. For the preparation of the combined aqueous extracts of Danshen and Sanqi (DS-SQ combination), the crude herbal powder of Danshen and Sanqi was combined in nine different (w/w) ratios (1 : 9, 2 : 8,3 : 7,…, 8 : 2,9 : 1), and proceeded to the identical extraction procedure as single extract.

Chemical standards for Danshen including sodium danshensu (DSS), salvianolic acid B (SB), salvianolic acid A (SA), tanshinone TIIA (TIIA), dihydrotanshinone I (DT), tanshinone I (TI), and cryptotanshinone (CT) and those for Sanqi including ginsenoside Rg1 (Rg1), ginsenoside Rg2 (Rg2), ginsenoside Rd (Rd), ginsenoside Rb1 (Rb1), and notoginsenoside R1 (NR1) were purchased from Chengdu Biopurify Phytochemicals Ltd. (Chengdu, China; purity >98%). The standards were verified via liquid chromatography-mass spectrometry. The standard stock solutions of these reference compounds were prepared in methanol and stored at 4°C until further use.

### 2.2. Cell Culture

The murine RAW264.7 macrophages cells were cultured at 37°C in DMEM (Life Technologies, Victoria, Australia) supplemented with 5% foetal bovine serum (FBS) (Life Technologies, Victoria, Australia), 1% GlutaMax, and 1% penicillin-streptomycin (Life Technologies, Victoria, Australia) in a humidified atmosphere containing 5% CO_2_ and 95% air.

### 2.3. NO Assay

The NO production stimulated by LPS in RAW264.7 cells was measured by its stable metabolite nitrite based on the Griess reaction [25]. Briefly, cells (density at 1 × 10^6^/mL) were seeded on 96-well cell culture plate (Corning® Costar®, Sigma, Australia) and incubated for 48 hours followed by pretreatments with individual DS or SQ extracts or DS-SQ combinations. After the cells were incubated for 2 hours, 50 ng/mL of LPS was added to the cells and coincubated for another 24 hours. LPS from* Escherichia coli* 0111:B4 purified by trichloroacetic acid extraction (LPS, Sigma, batch number 070M4018) was used to stimulate inflammatory mediators including NO, TNF, and MCP-1. After the LPS stimulation, 80 *μ*L of cells supernatant was collected and mixed with Griess reagent (1% sulfanilamide in 5% phosphoric acid and 0.1% N-1-naphthylethylenediamine dihydrochloride in Milli-Q water) for NO measurement. The other part of the cell supernatant was used for TNF and MCP-1 ELISA assay. The plate with mixed supernatant and Griess reagent was monitored under 540 nm.

### 2.4. TNF and MCP-1 ELISA Assays

The stored supernatants were analysed for TNF and MCP-1 synthesis using a commercial ELISA kit (Peprotech, Queensland, Australia) according to the manufacturer's instructions. The absorbance was measured at 410 nm. The concentrations of TNF and MCP-1 in the experimental samples were extrapolated from a standard curve.

### 2.5. (3-(4,5-Dimethylthiazol-2-yl)-2,5-diphenyltetrazolium bromide) (MTT) Assay

The cytotoxicities of the DS, SQ, and DS-SQ extracts were tested using MTT assay. MTT (0.5 mg/mL) was added to the cells and incubated for 4 hours. The supernatant was then discarded and replaced with the same amount of dimethyl sulfoxide (DMSO) (Sigma, Australia) and the optical density was measured using a microplate reader (BMG LABTECH FLUOstar OPTIMA, Mount Eliza, Victoria, Australia) at 510 nm. The density of formazan formed in control (medium with vehicle) cells was taken as 100% of cell viability.

### 2.6. Determination of Synergistic, Additive, or Antagonistic Interactions

Potential interactions of the combined extract were determined using the CI model based on Chou-Talalay's method [26]. Firstly, the concentration-response curves of the individual extract/compounds and their combinations in a fixed ratio pertaining to the bioassay were constructed and entered into CalcuSyn software 2.0 (Biosoft, USA). The combination index-fraction affected (CI-Fa) curve, isobologram figure, and the relevant statistics regarding the synergistic/antagonistic interactions were generated. The CI-Fa curve demonstrated the relationship between the CI value and the effective level on a certain biological target (i.e., the suppressive effect on NO). The CI values denoted synergism (CI < 1), additive effect (CI = 1), and antagonism (CI > 1). This curve exhibited the synergistic/antagonistic interaction at a particular effective range.

### 2.7. Statistical Analysis

All data were expressed as mean ± SEM (*n* ≥ 3). The statistical differences were analysed using one-way ANOVA followed by Tukey's Honestly Significant Difference (HSD) test. Values of *p* < 0.05 were considered statistically significant.

## 3. Results

### 3.1. Effects of DS, SQ, and DS-SQ on the Inhibition of Inflammatory Mediators Stimulated by LPS in RAW264.7 Cells

#### 3.1.1. Inhibitory Effects of DS, SQ, and DS-SQ on Nitrite Release

In the presence of LPS (50 ng/mL), a significant amount of nitrite production was detected and reached 215.56 ± 8.38 and 197.40 ± 3.36 *μ*g/mL in sterile water and DMSO vehicle solutions, respectively. A highly selective iNOS inhibitor dihydrochloride (1400 W) was used as the positive control in this model and showed a strong nitrite suppressive effect, with a half-maximal inhibitory concentration (IC_50_) value at 5.53 *μ*M.

**Figure 1:**
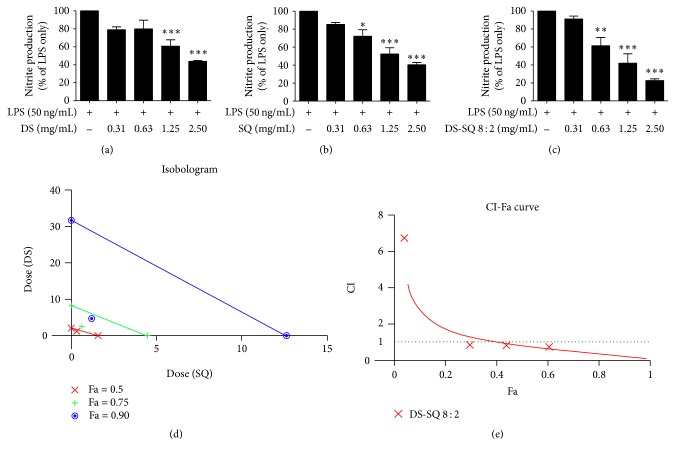
The suppressive effects of DS, SQ, and DS-SQ 8 : 2 on LPS-induced NO production are shown in (a), (b), and (c), respectively. (d) Isobologram curves of DS, SQ, and DS-SQ. (e) Combination index (CI) values were plotted as a function of fractional suppression of NO production (Fa) by “CalcuSyn” software. Dotted line is the reference line, where CI value is equal to 1; solid line represents CI values at different Fa. Fa values correspond to % suppression. ^*∗*^ *p* < 0.05, ^*∗∗*^ *p* < 0.01, and ^*∗∗∗*^ *p* < 0.001 as compared to LPS stimulation only by one-way ANOVA.

All tested single and combinational extracts of Danshen and Sanqi showed NO inhibitory effects in a dose-dependent manner (Figure 1) without the impairment of cell viability. At 2.5 mg/mL, DS and SQ significantly inhibited LPS-induced NO production in RAW264.7 cells by 56.08 ± 1.00% and 59.58 ± 2.42%, respectively. The IC_50_ values for DS and SQ in inhibiting NO were 2.19 and 2.06 mg/mL, respectively (Table 1). All DS-SQ combinations showed NO inhibitory effects in a dose-dependent manner, with IC_50_ values ranging from 0.34 to 2.98 mg/mL. It is noteworthy that the IC_50_ values for all DS-SQ combinations were lower than those of the single extracts except DS-SQ 9 : 1. DS-SQ 8 : 2 demonstrated the strongest NO inhibitory effect with the lowest IC_50_ value of 0.34 mg/mL, and at 2.5 mg/mL the NO generation was reduced by 77.29 ± 1.85% (*p* < 0.001).

#### 3.1.2. Inhibitory Effects of DS, SQ, and DS-SQ on TNF Release

**Figure 2:**
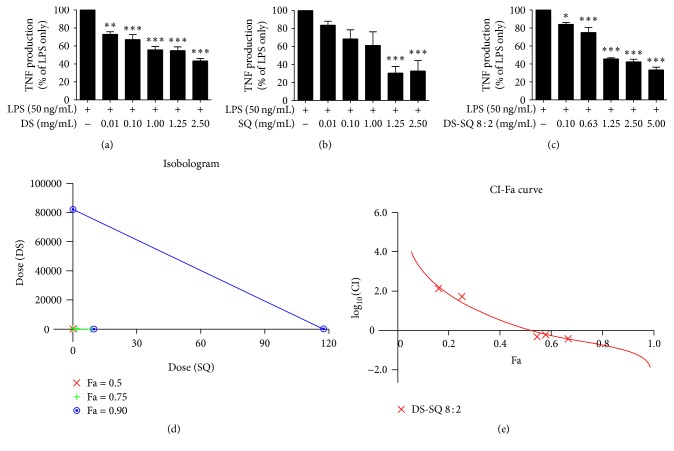
Dose-response curves of TNF production (%) versus concentration (mg/mL) for DS, SQ, and DS-SQ 8 : 2 on ELISA assays as shown in (a), (b), and (c). (d) Isobologram curves for DS, SQ, and DS-SQ 8 : 2 on TNF ELISA assay. (e) CI values versus DS-SQ 8 : 2 TNF inhibition effect (Fa). Solid line represents CI values at different Fa. Fa values correspond to % suppression. ^*∗*^ *p* < 0.05, ^*∗∗*^ *p* < 0.01, and ^*∗∗∗*^ *p* < 0.001 as compared to LPS stimulation only by one-way ANOVA.

In the presence of LPS (50 ng/mL), a significant amount of TNF production was detected and reached 870.08 ± 44.22 pg/mL in DMSO vehicle solution. The LPS-induced TNF production in RAW264.7 cells was suppressed by DS (0.01–2.5 mg/mL), SQ (1.25–2.5 mg/mL), and DS-SQ (0.1–5.0 mg/mL) extracts in a dose-dependent manner (Figure 2) with no cytotoxicity. DS and SQ inhibited TNF production by 66.38 ± 9.92% (*p* < 0.001) and 70.93 ± 0.33% (*p* < 0.001), respectively, at 2.5 mg/mL, with IC_50_ values of 1.43 and 1.71 mg/mL, respectively (Table 1). Similarly, all DS-SQ combinations showed TNF inhibitory effects in a dose-dependent manner, with IC_50_ values ranging from 0.61 to 1.29 mg/mL. These values were lower than of the DS and SQ individual extracts, suggesting stronger inhibitory effects by the combination. The inhibitory effects generated by DS-SQ 6 : 4 to 8 : 2 appeared to be relatively stronger than other combinations (IC_50_ from 0.61 to 0.85 mg/mL), with DS-SQ 7 : 3 (0.1−5 mg/mL) having the most potent effect with the lowest IC_50_ value (0.61 mg/mL). DS-SQ 8 : 2 (0.1−5 mg/mL), which produced the greatest NO inhibitory effect, also showed a marked effect on TNF (66.57 ± 2.93% at 2.5 mg/mL; IC_50_ = 0.85 mg/mL).

#### 3.1.3. Inhibitory Effects of DS, SQ, and DS-SQ on MCP-1 Release

**Figure 3:**
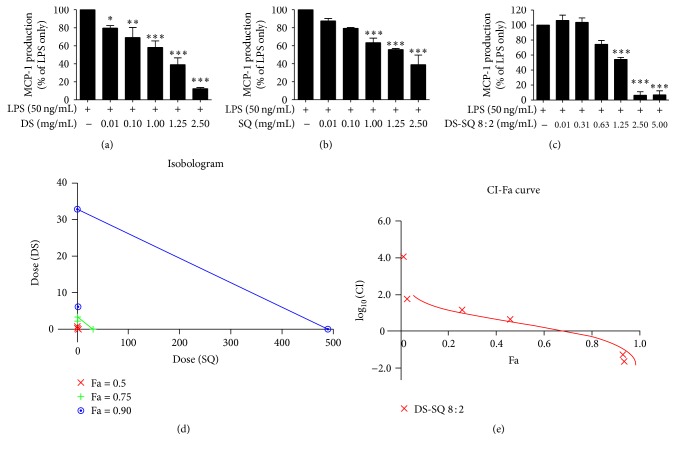
Dose-response curves of MCP-1 production (%) versus concentration (mg/mL) for DS, SQ, and DS-SQ 8 : 2 on ELISA assays as shown in (a), (b), and (c). (d) Isobologram curves for DS, SQ, and DS-SQ 8 : 2 on MCP-1 ELISA assay. (e) CI values versus DS-SQ 8 : 2 MCP-1 inhibition effect (Fa). Solid line represents CI values at different Fa. Fa values correspond to % suppression. ^*∗*^ *p* < 0.05, ^*∗∗*^ *p* < 0.01, and ^*∗∗∗*^ *p* < 0.001 as compared to LPS stimulation only by one-way ANOVA.

In the presence of LPS (50 ng/mL), a significant amount of MCP-1 production was detected and reached 2435.38 ± 156.45 pg/mL in DMSO vehicle solution. Individual DS (0.01−2.5 mg/mL) and SQ (0.01−2.5 mg/mL) extracts suppressed LPS-induced MCP-1 generation in a dose-dependent manner, with IC_50_ value at 1.39 and 1.87 mg/mL, respectively (Figure 3, Table 1). At a concentration of 2.5 mg/mL, DS and SQ inhibited MCP-1 production by 87.53 ± 1.14% (*p* < 0.01) and 71.37 ± 3.42% (*p* < 0.01), respectively. All DS-SQ combinations (except DS-SQ 2 : 8) demonstrated a greater MCP-1 suppressive effect than DS and SQ alone, with IC_50_ values ranging from 0.69 to 1.94 mg/mL. Relatively stronger effects were found in the DS-SQ combinations that have more DS (6 : 4 to 9 : 1), with DS-SQ 7 : 3 demonstrating the strongest effect (IC_50_ 0.69 mg/mL). DS-SQ 8 : 2 (0.1−5 mg/mL) also exerted a potent effect in the MCP-1 assay and produced an 89.75 ± 4.73% MCP-1 reduction at 2.5 mg/mL.

All inhibitory effects of DS, SQ and DS-SQ combinations on the above assays were not due to cytotoxicity.

#### 3.1.4. Optimised Ratio of DS-SQ Combinations in NO, TNF and MCP-1 Assays

Based on the above results from the NO, TNF, and MCP-1 assays (as shown in Table 2), DS-SQ 8 : 2 consistently produced low IC_50_ values in the suppression of the three inflammatory mediators (NO, TNF, and MCP 1) and, therefore, was selected as the optimal ratio for anti-inflammatory activity.

#### 3.1.5. Synergy Determination of DS-SQ 8 : 2 in Inhibiting NO, TNF, and MCP-1

Based on the dose-response curves of the DS, SQ, and DS-SQ 8 : 2 (Figures 1, 2, and 3), CI values at specific effect level (e.g., half-maximal effective dose, ED_50_) were calculated using “CalcuSyn” software with classical isobologram equation. Isobologram curves (Figures 1(d), 2(d), and 3(d)) and CI-Fa curves (Figures 1(e), 2(e), and 3(e)) were generated, so that CI values at different effect levels could be easily compared.

As shown in Figure 1(d), DS-SQ 8 : 2 isobologram produced a synergistic effect in inhibiting NO generation indicating that lower concentrations were required of the combination to reach the same effect level compared with that of the single extracts at ED_50_, ED_75_, and ED_90_. Moreover, this synergistic effect was confirmed by the CI-Fa curve of DS-SQ 8 : 2, with Fa value > 0.4 (40% of the NO inhibition). Thus, it was calculated that DS-SQ 8 : 2 exerted synergistic effects when its concentration was higher than 1.24 mg/mL (Figure 1(e)).

Similarly, as shown in Figure 2(d), the isobologram showed a synergistic interaction between DS-SQ 8 : 2 in inhibiting TNF at 90% inhibitory effect level (ED_90_). The CI-Fa curve in Figure 2(e) suggested that there was a synergistic effect when Fa value was greater than 0.523 for DS-SQ 8 : 2. The corresponding synergistic concentration for DS-SQ 8 : 2 was calculated to be above 1.89 mg/mL.

In Figure 3(d), the isobologram showed synergistic interactions for DS-SQ 8 : 2 in inhibiting MCP-1 at 75 and 90% inhibitory effect levels (ED_75_ and ED_90_). Furthermore, the CI-Fa curve revealed a biphasic effect: a synergistic effect was produced when the DS-SQ 8 : 2 concentrations were greater than 2.17 mg/mL and vice versa, indicating an antagonistic effect (Fa < 0.7) (Figure 3(e)).

In summary, the minimum concentrations of DS-SQ 8 : 2 to exert synergistic effects in inhibiting LPS-induced NO, TNF, and MCP-1 release were 1.24, 1.89, and 2.17 mg/mL, respectively (Table 3).

### 3.2. Inhibitory Effects of Bioactive Components of Danshen and Sanqi on Inflammatory Mediators

#### 3.2.1. Effects on NO Production

Most of the bioactive compounds tested demonstrated dose-dependent inhibition of NO production. SB (1–1000 *μ*M) and Rg1 (1–800 *μ*M), the two most abundant compounds of DS and SQ, respectively, produced a maximum reduction of NO generation by 72.16 ± 5.8% and 18.67 ± 3.77%, respectively, with an IC_50_ value of 108.30 *μ*M for SB (Table 4). By contrast, the relatively lipid-soluble compounds (DT, CT, T1, Rg2, and Rd) showed greater NO inhibition (IC_50_ ranging from 4.64 to 116.80) compared to their water-soluble counterparts (NR1, Rg1, Rb1, DSS, SA, and SB) as evidenced by significantly lower IC_50_ values (ranging from 26.02 to 144.10 *μ*M) (*p* < 0.5) (Table 4). DT (3.91–15.63 *μ*M) and Rd (10–100 *μ*M) were found to be the key compounds responsible for NO inhibition (95.16 ± 0.25% and 88.00 ± 2.14% at the maximum, with IC_50_ values at 4.64 and 7.89 *μ*M, resp.) and were comparable to those of the positive control dihydrochloride (IC_50_ = 5.53 *μ*M).

#### 3.2.2. Effects on TNF Production

Similar to the results in the NO assay, the relatively lipid-soluble compounds (CT, DT, TI, Rb2, Rg2, and Rd) caused dose-dependent inhibition of LPS-induced TNF production, with the IC_50_ ranging from 1.98 to 28.64 *μ*M without cytotoxicity (Table 4). With the exception of Rb1, the inhibitory effects of the relatively water-soluble compounds did not reach 50% even after the concentration was raised to 1000 *μ*M. CT (10–31.25 *μ*M) and Rd (0.1–200 *μ*M) were found to be the most potent compounds in inhibiting TNF production (93.28 ± 9.24% and 86.84 ± 1.61% at the maximum), with IC_50_ values at 1.98 and 9.79 *μ*M, respectively. DT (20–31.25 *μ*M) exerted a potent inhibitory effect (85.70 ± 2.49% at the maximum).

#### 3.2.3. Effects on MCP-1 Production

In the MCP-1 assay, only CT, DT, and Rd caused inhibitory effects on MCP-1 generation, with IC_50_ values of 11.32, 13.97, and 36.00 *μ*M, respectively. No cytotoxicity was observed. Other tested compounds did not show any significant MCP-1 inhibitory effect (*p* > 0.05). Among the three bioactive compounds, DT (20 *μ*M) and Rd (100 *μ*M) produced potent inhibitory effects (82.26 ± 7.91% and 56.57 ± 5.98%, resp.).

The concentrations for all the tested bioactive compounds in the NO, TNF, and MCP-1 bioassays were within their safe dosage for RAW264.7 cells.

#### 3.2.4. Interactions between DT and Rd in Inhibiting NO, TNF, and MCP-1 Release

**Figure 4:**
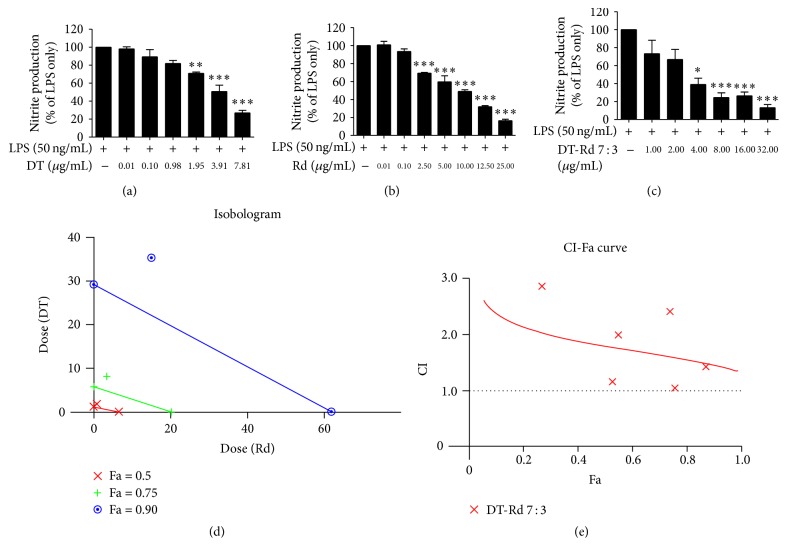
Dose-effect curves for DT, Rd, and DT-Rd (7 : 3) on NO assay as shown in (a), (b), and (c). (d) Isobologram curves for DT, Rd, and DT-Rd (7 : 3) on NO. (e) CI values versus NO inhibition effect (Fa) by “CalcuSyn” software. Dotted line is the reference line, where CI value is equal to 1; solid line represents CI values at different Fa. Fa values correspond to % suppression. ^*∗*^ *p* < 0.05, ^*∗∗*^ *p* < 0.01, and ^*∗∗∗*^ *p* < 0.001 as compared to LPS stimulation only by one-way ANOVA.

**Figure 5 fig5:**
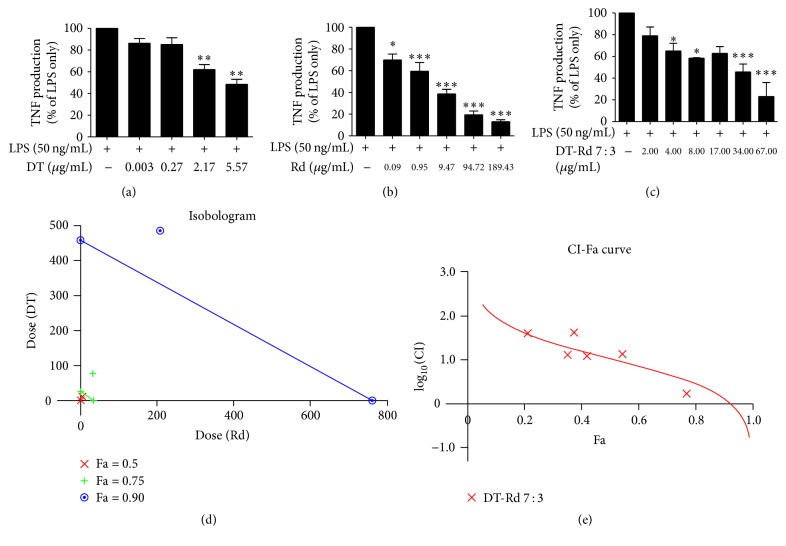
Dose-effect curves for DT, Rd, and DT-Rd (7 : 3) on TNF assay as shown in (a), (b), and (c). (d) Isobologram curves for DT, Rd, and DT-Rd (7 : 3) on TNF. (e) CI values versus TNF inhibition effect (Fa) by “CalcuSyn” software. Solid line represents CI values at different Fa. Fa values correspond to % suppression. ^*∗*^ *p* < 0.05, ^*∗∗*^ *p* < 0.01, and ^*∗∗∗*^ *p* < 0.001 as compared to LPS stimulation only by one-way ANOVA.

**Figure 6:**
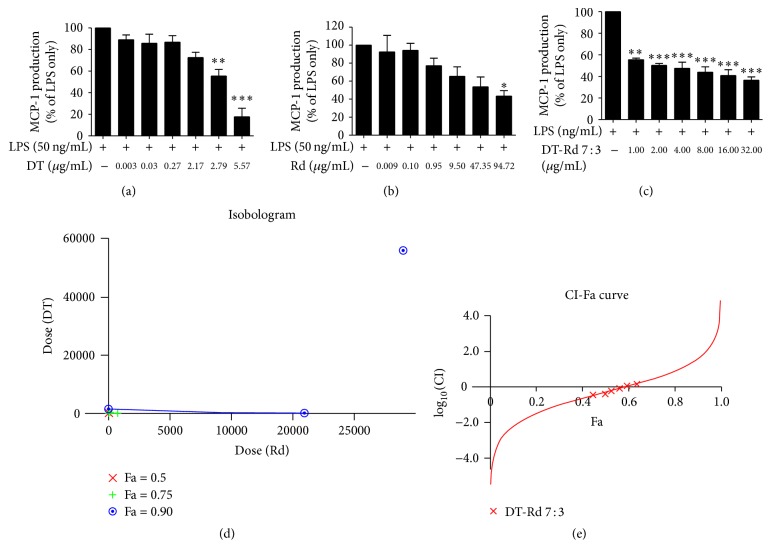
Dose-effect curves for DT, Rd, and DT-Rd (7 : 3) on MCP-1 assay as shown in (a), (b), and (c). (d) Isobologram curves for DT, Rd, and DT-Rd (7 : 3) on MCP-1. (e) CI values versus MCP-1 inhibition effect (Fa) by “CalcuSyn” software. Solid line represents CI values at different Fa. Fa values correspond to % suppression. ^*∗*^ *p* < 0.05, ^*∗∗*^ *p* < 0.01, and ^*∗∗∗*^ *p* < 0.001 as compared to LPS stimulation only by one-way ANOVA.

DT from Danshen and Rd from Sanqi were found to be the most potent bioactive compounds in inhibiting LPS-induced NO, TNF, and MCP-1 generation (Figures 4 [Fig fig5]–6, Table 4) and thus were evaluated for their potential synergic interactions.

The IC_50_ values for the DT-Rd combinations in various ratios (1 : 9,2 : 8,…, 8 : 2,9 : 1 (w : w)) ranged from 2.08 to 36.65 *μ*g/mL (Table 5). DT-Rd combinations with the ratio of 1 : 9 to 5 : 5 showed significantly higher IC_50_ values (12.47–36.65 *μ*g/mL) in inhibiting NO than other combinations (IC_50_ values 1.28–7.36 *μ*g/mL) (*p* < 0.05). The NO inhibitory effects of the DT-Rd combinations became stronger when the DT proportion increased, with DT-Rd ratios from 7 : 3 to 9 : 1 showing comparable IC_50_ values as that of DT on its own.

A similar trend was found for TNF inhibition; the combinations with a higher proportion of DT (7 : 3 to 9 : 1) produced greater inhibitory effects (IC_50_ values ranging from 2.01 to 9.10 *μ*g/mL) than those with a lower DT ratio (IC_50_ values ranging from 9.41 to 35.56 *μ*g/mL). Among them, the DT-Rd 7 : 3 combination showed the greatest inhibitory effect, with an IC_50_ value of 2.01 *μ*g/mL, which was significantly lower than that of DT and Rd on their own.

In the MCP-1 assay, all DT-Rd combinations showed significantly higher IC_50_ values (12.21–92.15 *μ*g/mL) than that of DT (IC_50_ = 3.89 *μ*g/mL) on its own. In spite of this, DT-Rd (7 : 3) exhibited significantly lower IC_50_ (12.21 *μ*g/mL) than Rd on its own (IC_50_ = 34.10 *μ*g/mL), suggesting the greatest inhibitory effect among all DT-Rd combinations.

#### 3.2.5. Synergy Determination of DT-Rd Combination

The DT-Rd 7 : 3 combination consistently showed greater inhibitory effects on NO, TNF, and MCP-1 production in the assays (IC_50_ values were 2.08, 2.01, and 12.21 *μ*g/mL, resp.) when compared with other combinations and was therefore considered to be the optimised ratio for synergistic interactions.

Both the isobologram and the CI-Fa curve suggested that the DT-Rd 7 : 3 combination exhibited an antagonistic inhibitory effect on LPS-induced NO generation (CI value was at 1.79 at Fa = 0.5) (Figures 4(d) and 4(e)). Similar antagonistic effect was observed in the TNF assay when the concentration of DT-Rd (7 : 3) was lower than 103.92 *μ*g/mL (Fa > 0.744) (Figures 5(d) and 5(e)). A slight synergistic effect on MCP-1 generation was shown when Fa was lower than 0.584. The interaction tended to be additive when Fa was increased to 0.6. Antagonism was observed and predicted to be stronger when Fa was higher than 0.6 (the concentration for DT-Rd was calculated to be higher than 12.65 *μ*g/mL).

## 4. Discussion

The anti-inflammatory effects of Danshen and Sanqi as single herbs have been well demonstrated in previous* in vitro* and* in vivo* studies and were confirmed in the current study. In addition, our results demonstrated synergistic effects of the Danshen-Sanqi combinations on the aforementioned key anti-inflammatory events using the CI and isobologram models. To the best of our knowledge, this is the first report of the synergistic effects of a Danshen-Sanqi combination on anti-inflammatory pathway associated with vascular diseases.

Our results showed that the aqueous extract of Danshen significantly inhibited LPS-induced NO, TNF, and MCP-1 production in RAW264.7 cells, which is consistent with the previous* in vitro* and* in vivo* findings. The inhibitory effects of Danshen have been suggested to be associated with the attenuation of mRNA expressions of the inflammatory mediators [27, 28]. Further, our study has demonstrated for the first time that lipid-soluble components of Danshen (e.g., DT, T1, and CT) produced a greater inhibitory effect on NO production than their water-soluble counterparts (e.g., DSS, SA, and SB). Specifically, DT was found to exert a comparable inhibitory effect on NO to that of dihydrochloride, a specific iNOS inhibitor, highlighting its anti-inflammatory potential as a single chemical entity. The anti-inflammatory activities of tanshinones reported in this study are compatible with the findings of previous studies. It was demonstrated that lipid-soluble extracts of Danshen were capable of inhibiting the LPS-induced gene and protein expression of iNOS, TNF, IL-1*β*, and IL-6 in macrophages via blocking NF-*κ*B activation [18, 19]. It was also previously reported that CT downregulated the expression of proinflammatory enzymes, including COX-2 and iNOS [18]. Many* in vitro* studies suggest that TIIA is a key bioactive component of Danshen responsible for its anti-inflammatory activities through suppressing LPS-stimulated NO, IL-1*β*, IL-6, and MCP-1 expressions [29–31].

In this study, the Sanqi extract also demonstrated significant inhibitory effects on NO, TNF, and MCP-1 production which has been suggested to be associated with downregulating the expression of COX-2 mRNA and blockage of NF-*κ*B via LXR*α* [32]. Ginsenosides from Sanqi were found to be the key components contributing to its anti-inflammatory effects via multiple mechanisms [33]. Rb1 and Rb2 have been suggested to block TNF production and to attenuate NO and PGE2 via repression of NF-*κ*B activation signals. Rg1 and Re have been shown to inhibit NO and TNF production via ERK and JNK pathways, and Rb2 was shown to target cAMP PDE [33]. It was demonstrated that the anti-inflammatory mechanism of Rd was associated with inhibiting iNOS and COX-2 expression [33]. Our results support these findings and found Rd as the key ginsenoside compound (lowest IC_50_ value) responsible for the anti-inflammatory properties of Sanqi [33].

Danshen and Sanqi have been widely used in combination clinically for the treatment of various cardiovascular diseases. This combinational approach is common in TCM practice based at least partially on an assumption that various components of herbs in complex herbal formulations can interact to produce synergistic effects leading to better therapeutic outcomes. In TCM, the ratio (dosage) of herbal ingredients is tailored to each patient in order to reach a desirable clinical outcome based on syndrome-differentiation treatment strategy. As recorded in a classic TCM textbook, Danshen should be used at a higher proportion in the DS-SQ combination in the early stages of cardiovascular disease when pathological changes of the organs have not occurred, whereas the proportion of Sanqi should be increased at the later stage of the disease [34]. However, the scientific evidence to support this theory is limited. In a previous study, a higher proportion of Danshen in the combination (10 : 3–10 : 7) was also shown to be better than Danshen or Sanqi alone for improving myocardial ischaemia and inhibiting platelet adhesion and aggregation* in vivo* [35]. It was assumed that this enhanced biological activity of the combination was associated with its altered chemical composition during the mixed preparation. Interestingly, an analytical study by Zeng et al. supported this assumption, which showed that the extraction yield of the chemical constituents of Danshen was increased in the codecocted Danshen and Sanqi mixtures, with the ratio of 5 : 3 yielding the highest quantity of chemical constituents from Danshen [36]. This suggests that the amount of the components in Danshen was increased in the presence of Sanqi in the mixture. This may be due to Sanqi providing an environment which solubilises the Danshen compounds due to the hydrophilic/hydrophobic nature of the saponins. This is the first study that systematically evaluated the synergy of Danshen and Sanqi combinations on three anti-inflammatory pathways. We found that the DS-SQ 8 : 2 combination produced the strongest anti-inflammatory effects. Moreover, the combination reached synergistic effects (CI < 1) in inhibiting LPS-induced NO, TNF, and MCP-1 when the concentration was greater than 1.24 mg/mL, 1.89 mg/mL, and 2.17 mg/mL, respectively. The optimal ratio of Danshen-Sanqi at 8 : 2 for the anti-inflammatory activity supports the traditional DS-SQ combination for the early stage of cardiovascular disease whereby a higher proportion of Danshen is used [34]. In addition, these results have provided strong evidence to support synergistic interactions in this 2-herb combination. A previous study applied systems biology to explain the synergistic action of Danshen-Sanqi at the molecular level. It was discovered that Danshen, when used in a higher proportion, was the key player, and its chemical compounds bound to the main targets involved in cardiovascular disease. However, the presence of Sanqi provided an overall stronger outcome by serving as an “assistant” whereby its chemical compounds bound to other associated targets [37]. Therefore, this reflects the traditional theory of the combination whereby Danshen is the primary herb at a higher dosage and Sanqi is the assistant herb at a lower dosage.

In terms of a pharmaceutical combination, it is interesting to note that although DT-Rd 7 : 3 produced the most prominent inhibitory effects on NO, TNF, and MCP-1 release, an antagonistic relationship between the two components was identified. This suggests that combining two potent compounds may not necessarily engage a stronger effect and, in fact, may lead to a reduced response (antagonism) compared with individual effects. One possibility is that single, active compounds may compete for/saturate the same cellular receptor/metabolic pathways, leading to lower than expected cellular responses than predicted.

The anti-inflammatory effects of Danshen-Sanqi combinations have been demonstrated with* in vitro* models in this study. However, we recognise that the cellular assays used in this study serve as a preliminary screening method. Whether the Danshen-Sanqi 8 : 2 combination can be applied to a broader range of inflammatory mediators and other* in vitro*/*in vivo*/clinical systems is yet to be determined. Additionally, in-depth investigation on Danshen-Sanqi 8 : 2 such as signaling pathways, target protein, and gene expressions may need to be examined in future studies.

## 5. Conclusions

The results showed that the Danshen-Sanqi combination with a ratio of 8 : 2 synergistically enhanced their inhibitory activities on LPS-induced NO, TNF, and MCP-1 in RAW264.7 cells. Additionally, the relatively nonpolar compounds of the herbs demonstrated stronger effects than their polar counterparts, suggesting a greater role of the lipid-soluble components in the anti-inflammatory activities of the two herbal extracts. DT and Rd, two lipophilic compounds derived from Danshen and Sanqi, respectively, were found to be the most potent compounds in inhibiting the LPS-induced NO, TNF, and MCP-1 production. However, DT-Rd combinations produced antagonistic interactions. In summary, this study provides early evidence to support the traditional use of the Danshen-Sanqi combination.

## Figures and Tables

**Table 1:** IC_50_ values of DS, SQ, and DS-SQ in inhibiting NO, TNF, and MCP-1 expressions induced by LPS on RAW264.7 cells.

Danshen : Sanqi	IC_50_ (mg/mL)		
	NO	TNF	MCP-1
0 : 10	2.06	1.71	1.87
1 : 9	1.10	0.72	0.86
2 : 8	1.39	1.29	1.94
3 : 7	1.27	0.84	1.68
4 : 6	1.26	1.07	1.72
5 : 5	1.59	1.22	1.34
6 : 4	1.11	0.80	0.99
7 : 3	1.40	0.61	0.69
*8 : 2*	*0.34*	*0.85*	*0.97*
9 : 1	2.98	1.02	1.49
10 : 0	2.19	1.43	1.39

**Table 2:** Inhibitory effects of DS, SQ, and DS-SQ 8 : 2 on LPS-induced NO, TNF, and MCP-1 production in RAW264.7 cells at 2.5 mg/mL.

Tested extracts	Inhibition of LPS-induced proinflammatory mediators (% ± SEM)		
	NO	TNF	MCP-1
DS	56.08 ± 1.00	66.38 ± 9.92	87.53 ± 1.14
SQ	59.58 ± 2.42	70.93 ± 0.33	71.37 ± 3.42
DS-SQ 8 : 2	77.29 ± 1.85	66.57 ± 2.93	89.75 ± 4.73

**Table 3:** Dose range of DS-SQ 8 : 2 and DT-Rd 7 : 3 which showed synergistic effect (CI < 1) calculated by CalcuSyn.

Synergy dose range			
Combination	NO assay	TNF ELISA assay	MCP-1 ELISA assay
DS-SQ 8 : 2	>1.24 mg/mL	>1.89 mg/mL	>2.17 mg/mL
DT-Rd 7 : 3	>22.85 *μ*g/mL	>2.22 *μ*g/mL	<0.053 *μ*g/mL

Data was obtained from CalcuSyn.

**Table 4:** IC_50_ values of the key bioactive compounds of Danshen and Sanqi in NO, TNF, and MCP-1 assay.

	DS compounds	IC_50_ (*μ*M)		
		NO	TNF-*α*	MCP-1
Relatively water-soluble components	DSS	144.10	NA	NA
	SA	90.23	NA	NA
	SB	108.30	NA	NA

Relatively lipid-soluble components	CT	116.80	1.98	11.32
	DT	4.64	24.25	13.97
	T1	5.45	7.627	NA
	TIIA	NA	NA	NA

	SQ compounds	IC_50_ (*μ*M)		
		NO	TNF-*α*	MCP-1

Relatively water-soluble components	NR1	92.05	NA	NA
	Rg1	NA	NA	NA
	Re	26.02	NA	NA
	Rb1	53.9	71.36	NA

Relatively lipid-soluble components	Rb2	134.90	28.44	NA
	Rg2	66.41	28.64	NA
	Rd	7.89	9.79	36.00

NA: the maximum inhibitory effect less than 50%.

**Table 5:** IC_50_ value of DT-Rd combination in NO, TNF, and MCP-1 ELISA assays.

DT-Rd ratio	IC_50_ value (*μ*g/mL)		
	NO	TNF-*α*	MCP-1
0 : 10	7.47	12.06	34.10
1 : 9	33.72	31.20	48.50
2 : 8	36.65	35.56	56.35
3 : 7	26.66	33.46	92.15
4 : 6	25.46	25.67	29.03
5 : 5	12.47	9.41	34.47
6 : 4	7.36	31.03	47.13
*7 : 3*	*2.08*	*2.01*	*12.21*
8 : 2	2.53	2.46	16.06
9 : 1	1.28	9.10	14.52
10 : 0	1.29	87.14	3.89
